# Effect of Bee Bread on Corticosterone Level in Rat Dams Exposed to Gestational Heat Stress

**DOI:** 10.21315/tlsr2023.34.3.8

**Published:** 2023-09-30

**Authors:** Nur Akmar Nadhirah Mohd Nor, Asmad Kari, Mohd Nizam Haron, Connie Fay Komilus

**Affiliations:** School of Animal Science, Aquatic Science and Environment, Faculty of Bioresources and Food Industry, Universiti Sultan Zainal Abidin 22200 Besut, Terengganu, Malaysia

**Keywords:** Heat Stress, Corticosterone, Adrenal Gland, Female Reproduction, Bee Bread, Tekanan Haba, Kortikosteron, Kalenjar Adrenal, Reproduksi Betina, Roti Lebah

## Abstract

Environmental temperature rises are powerful stimuli that can alter both the sympathetic nervous system and the hypothalamic-pituitary-adrenocortical axis (HPA). Heat stress has been shown to harm pregnancy outcomes such as causing spontaneous abortion, low birth weight, growth retardation and stillbirth. Supplementation of bee bread in pregnant rats under heat stress exposure has been shown to improve the pregnancy outcomes. However, whether supplementation of bee bread during heat stress exposure may also reduce the level of the stress hormone, corticosterone has yet been reported. Therefore, this study aims to determine the effect of bee bread on corticosterone level, progesterone level, oestradiol level and zonation of the adrenal cortex of pregnant rats under heat stress exposure. Pregnant rats were randomly categorised into four groups (*n* = 6): Control (C: standard feeding), Treatment 1 (T1: 0.5 g bee bread/kg body weight/day), Treatment 2 (T2: standard feeding with heat exposure), and Treatment 3 (T3: 0.5 g bee bread/kg body weight/day with heat exposure). Bee bread (0.5 g/kg body weight/day) was force-fed to pregnant rats through oral gavage beginning on day 0 of pregnancy and continuing until delivery. Heat stress was generated experimentally by putting both T2 and T3 rats in an egg incubator for 45 min daily at a temperature of 43°C till delivery. On a postnatal Day 21, dams were euthanised to assess serum corticosterone, progesterone, oestradiol levels and adrenal gland histology. Rats in the T2 group had a significantly (*P* < 0.05) increase in the zona fasciculata thickness (94.95 ± 1.55 μm) and higher corticosterone levels (49.57 ± 1.57 ng/mL) compared with control. However, supplementation of bee bread during heat stress was able to show an improvement in adrenal zona fasciculata thickness by decreasing to 79.89 ± 3.08 μm and corticosterone level reduced to 35.31 ± 1.73 ng/mL significantly (*P* < 0.05). Therefore, these findings may imply that bee bread is effective as a neutralizer in lowering the production of stress hormone.

## Abstrak

Peningkatan suhu persekitaran adalah rangsangan kuat yang boleh mengubah kedua-dua sistem saraf simpatetik dan paksi hipotalamus-pituitari-adrenokortikal (HPA). Tekanan haba telah terbukti membahayakan hasil kehamilan seperti pengguguran spontan, berat lahir rendah, terencat pertumbuhan dan kelahiran mati. Suplemen roti lebah pada tikus hamil di bawah pendedahan tekanan haba telah menunjukkan pemulihan dalam hasil kehamilan. Walau bagaimanapun, sama ada suplemen roti lebah semasa pendedahan tekanan haba juga boleh mengurangkan tahap hormon tekanan, kortikosteron masih belum dilaporkan. Oleh itu kajian ini bertujuan untuk menentukan kesan roti lebah terhadap paras kortikosteron, paras progesteron, paras estradiol dan pengezonan korteks adrenal tikus hamil di bawah pendedahan tekanan haba. Tikus hamil secara rawak dikategorikan kepada empat kumpulan (*n* = 6): Kawalan (C: pemakanan standard), Rawatan 1 (T1: 0.5 g roti lebah/kg berat badan/hari), Rawatan 2 (T2: pemakanan standard dengan pendedahan haba), dan Rawatan 3 (T3: 0.5 g roti lebah/kg berat badan/hari dengan pendedahan haba). Roti lebah (0.5 g/kg berat badan/hari) telah diberi makan secara paksa kepada tikus hamil melalui gavaj oral bermula pada hari 0 kehamilan dan berterusan sehingga bersalin. Tekanan haba dijana secara eksperimen dengan memasukkan kedua-dua tikus T2 dan T3 ke dalam inkubator telur selama 45 minit setiap hari pada suhu 43°C sehingga kelahiran. Pada hari ke-21 selepas kelahiran, ibu tikus telah dikorbankan untuk menilai paras kortikosteron, progesteron, estradiol dan histologi kelenjar adrenal. Tikus dalam kumpulan T2 mempunyai peningkatan ketara (*P* < 0.05) dalam ketebalan zona fasciculata (94.95 ± 1.55 μm) dan tahap kortikosteron yang lebih tinggi (49.57 ± 1.57 ng/mL) berbanding dengan kawalan. Walau bagaimanapun, suplemen roti lebah semasa tekanan haba telah menunjukan kesan positif dengan pengurangan ketebalan zona fasciculata (79.89 ± 3.08 μm) dan hormon kortikosteron (35.31 ± 1.73 ng/mL) secara ketara (*P* < 0.05). Oleh itu, penemuan ini mungkin membayangkan bahawa roti lebah berkesan sebagai peneutral dalam menurunkan pengeluaran hormon tekanan.

**Highlights**

- Bee bread is a nutritious, health-promoting food, rich in antioxidants, antibacterial, anticancer and hepatoprotective properties that is also packed with essential nutrients, such as carbohydrates, lipids, proteins, fatty acids and minerals.
- Rats in the T2 (heat stress) group had a significantly (*P* < 0.05) increase in the zona fasciculata thickness and higher corticosterone levels compared with control.
- Supplementation of 0.5 g bee bread during heat stress was able to reduce the zona fasciculate thickness and corticosterone level significantly compared with rats under heat stress exposure.

## INTRODUCTION

In both clinical and experimental studies, heat stress has been shown to have a negative impact on pregnancy outcomes such as spontaneous abortion, low birth weight, growth retardation and stillbirth (Schifano *et al*. 2016; Arroyo *et al*. 2016; Auger *et al*. 2017; Ha, Liu, Zhu, Kim, Sherman & Mendola 2017; Ha, Liu, Zhu, Kim, Sherman, Grantz & Mendola 2017). The precise mechanism of heat stress causing problems with pregnancy outcomes is still unclear. According to Sadek *et al*. (2021), both the hypothalamic-pituitary-adrenocortical (HPA) and sympatho-adrenomedullary axes are associated with maintaining homeostasis during stress. Elevated environmental rise are powerful stimuli that can alter both the sympathetic nervous system and the hypothalamic-pituitary-adrenocortical axis (HPA) (Huh *et al*. 2021). Elevated plasma corticosterone levels have previously been reported in heat-stressed broilers due to hypothalamic-pituitary-adrenal axis activation (Quinteiro-Filho *et al*. 2012; Xu *et al*. 2018). There is scientific proof that exposure to 8 h of heat stress at 32°C for 7 days increased adrenal weight in male rats while also increasing adrenocorticotrophic hormone (ACTH) and corticosterone levels (Wang *et al*. 2015).

Moreover, the activation of HPA axis has a strong inhibitory effect on hypothalamic-pituitary-gonadal axis (HPG) as reported by Valsamakis *et al*. (2019). According to El-Ratel *et al*. (2020), the high ambient temperature was found to have an impact on female reproductive hormones, resulting in lower levels of LH, FSH, progesterone and estradiol. Furthermore, a study by Fotsing *et al*. (2016) found that chronic immobilisation stress caused a decrease in uterus weight in rats due to a lack of oestrogen and progesterone, which are required for uterus development suggesting that activation of HPA axis may disturb the female reproduction process.

Bee bread is one of the bee products with antioxidants, antibacterial, anticancer, and hepatoprotective characteristics (Kieliszek *et al*. 2018). It is also considered a well-balanced diet since it contains carbs, lipids, proteins fatty acids and trace minerals (Urcan *et al*. 2017; Tomás *et al*. 2017; Kieliszek *et al*. 2018; Belina-Aldemita *et al*. 2019; Bakour *et al*. 2022). Bee bread also contains necessary amino acids that humans are unable to produce (da Silva *et al*. 2014; Donkersley *et al*. 2017). Recently, supplementation of bee bread in pregnant rats under heat stress exposure has shown a recovery in pregnancy outcomes such as an increase in litter size, increase in foetal birth weight and reduction in the percentage of embryo resorption when compared with heat-stressed dams (Nor *et al*. 2021). Furthermore, a previous study by Haron *et al*. (2014) reported that supplementation of honey during stress was able to counteract the effect of stress and reduced the corticosterone level that leading to an improvement in pregnancy outcomes. However, whether supplementation of bee bread during heat stress exposure may also reduce the level of the stress hormone, corticosterone has yet been reported.

Therefore, this study aims to determine the effect of bee bread on corticosterone level, progesterone level, oestradiol level and zonation of the adrenal cortex of pregnant rats under heat stress exposure.

## MATERIALS AND METHODS

### Experimental Design

Twenty-four (24) eight-week-old sexually developed female Sprague Dawley rats were used for this study. The rats were housed separately in a cage with clean and absorbent bedding, commercial pellet food, water ad libitum, and were kept at a temperature of 20°C–24°C in a 12-hour light/dark cycle. This study protocol was approved by the UniSZA Animal and Plant Research Ethics Committee (UAPREC/04/043).

The oestrous regularity was first checked by taking the vagina fluid smear. This procedure was repeated for 10 days consecutively. Using a blunt-ended pipette, the vagina was flushed with 0.9% normal saline. Following that, a little quantity of the cell solution was evacuated onto a labelled glass slide before being viewed under a light microscope at 100× and 400× magnifications. The proestrous or oestrous phase of the cycle was determined once the regularity of the cycle is obtained. The female rats were then mated overnight with proven fertile male rats (males who have achieved mature sexual age and have successfully mated before) to induce conception. Each female rat was rechecked the next morning to detect the presence of sperm, and a positive sperm smear was designated as day 0 of pregnancy (Organisation for Economic Co-operation and Development [OECD] 2008).

Pregnant rats were randomly categorised into four groups (*n* = 6): Control (C: standard feeding), Treatment 1 (T1: 0.5 g bee bread/kg body weight/day), Treatment 2 (T2: standard feeding with heat exposure), and Treatment 3 (T3: 0.5 g bee bread/kg body weight/day with heat exposure). All treatment group rats received the treatment once daily in the morning until delivery. Bee bread (0.5 g/kg body weight/day) was force-fed to pregnant rats through oral gavage beginning on day 0 of pregnancy and continuing until delivery. The bee bread samples utilised in this investigation were the same as one of the bee bread samples tested for nutritional value by Othman *et al*. (2019). To make the administration of the bee bread simpler, it was diluted with 1.0 mL distilled water. To guarantee the exact intestinal administration of bee bread, the dilution was administered through oral gavage. The bee bread dosages were calculated based on human intake, which was one tablespoon daily or roughly 30 g/60 kg body weight (Kieliszek *et al*. 2018). Rats in C and T2 groups were given 1.0 mL of distilled water daily to experience the same force-feeding procedure as the T1 and T3 groups. Heat stress was generated experimentally by putting both T2 and T3 rats in an egg incubator (M&M Ternak) for 45 min daily at a temperature of 43°C till delivery. This procedure was modified from Mohd Nor and Haron (2018) study’s temperature and duration. After delivery, each dam was sacrificed via cervical dislocation (American Veterinary Medical Association [AVMA] 2013). Blood was drawn directly from the inferior vena cava to obtain serum, which was later used to determine the level of corticosterone, progesterone and estradiol hormone. The dam’s adrenal glands were also removed for histopathological examination.

### Assay of Hormone Level

Serum corticosterone, progesterone, and oestradiol levels were measured by using an enzyme-linked immunosorbent assay kit from Elabscience. All samples and reagents for ELISA were brought to room temperature prior to performing the assay. An aliqout of 50 μL standard and samples were added to each well. Another 50 μL of Biotinylated Detection antibory (Ab) were immediately added to each well. The wells were incubated for 45 min at 37°C. After being incubated, the contents of the wells were aspirated and rinsed with 350 μL of wash buffer per well for three times. The residual droplets were removed. Next, 100 μL horseradish peroxidase (HRP) conjugate working solution were added to each well. Then, the mixture was mixed thoroughly for 10 s before incubating for 30 min at room temperature. Following incubation, the well contents were vigorously shaken, and the washing procedure was carried out five more times. Each well received 90 μL of the substrate reaction, which was then incubated at room temperature for 15 min. The enzymatic reaction was stopped by adding 50 μL of stop solution to each well. Within 10 min of adding the stop solution, the plate was read at 450 ± 10 mm using an Ultra Microplate Reader.

### Histological Assessment of Adrenal Gland

**Figure 1:**
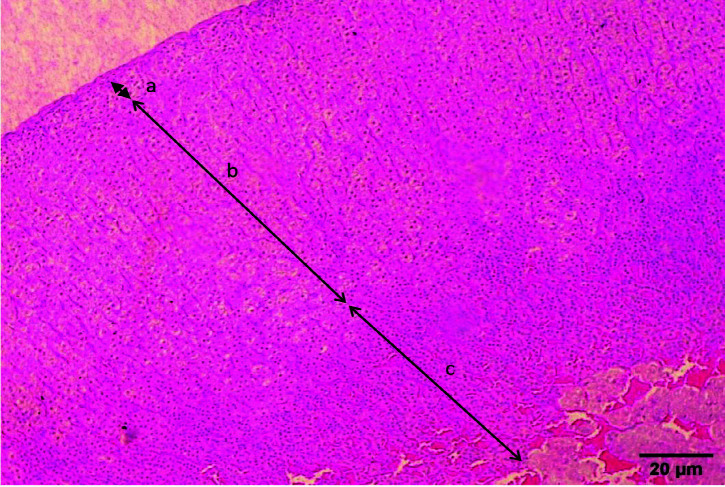
Light microscope feature of a histological section of rat adrenal gland, (a) Zona Glomerulosa, (b) Zona Fasciculata, (c) Zona Reticularis. (H&E staining, magnification of 400x).

The adrenal glands of the rats were taken immediately after they were slaughtered and submerged in 10% formalin for 24 h. After 24 h, the adrenal gland was cut with a sharp surgical blade and put inside a histology cassette before being dried and embedded in paraffin. On a microtome, solidified paraffin-embedded tissues were sliced into 4 μm thick slices. The section on the slides was stained using standard haematoxylin and eosin (H&E) staining methods ((National Society for Histotechnology [NSH] 2001). The thickness of the zona glomerulosa, zona fasciculata and zona reticularis was evaluated using the LAZ X image analyzer. The thickness was measured in two distinct locations of the adrenal gland, as illustrated in the histological section (Fig. 1) and was represented as the average mean of the two measurements (Haron *et al*. 2014).

### Statistical Analysis

The statistical analysis was performed using Minitab software. All normal distribution data were analysed using one way ANOVA, followed by Tukey’s post hoc test, and the results were expressed as mean ± SEM. Statistical significance was accepted at *P* < 0.05.

## RESULTS AND DISCUSSION

### Hormonal Level

**Figure 2:**
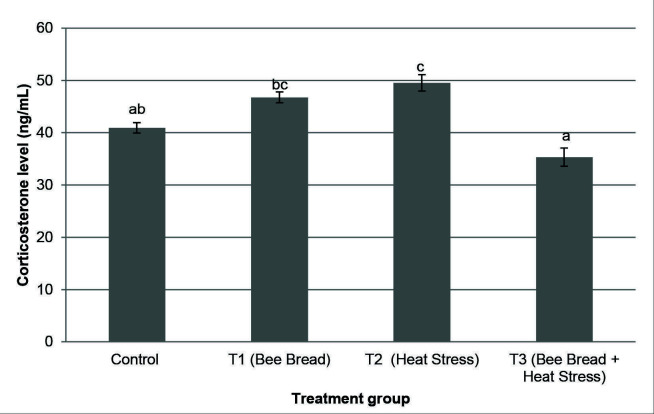
The corticosterone level (mean ± SEM) in different treatment groups which are Control (C: standard feeding treatment), Treatment 1 (T1: Bee bread), Treatment 2 (T2: Heat stress) and Treatment 3 (T3: Bee bread + Heat stress). Significant differences were determined by one-way ANOVA followed by Tukey’s post hoc test with *P* < 0.05. Different superscript (a, b, c) indicates a significant difference between the treatment groups. ^a^ indicates significant difference between C and T2; ^b^ indicates significant differences between T1 and T3 and ^c^ indicates significant difference between T2 and T3.

Fig. 2 depicts the results of all treatment groups’ serum corticosterone levels. When compared to the control and T3 groups, the T2 group had substantially (*P* < 0.05) greater corticosterone levels (49.57 ± 1.57 ng/mL). The corticosterone level in the T3 group, on the other hand, was significantly (*P* < 0.05) lower (35.31 ± 1.73 ng/mL) than in the T1 and T2 groups.

**Figure 3:**
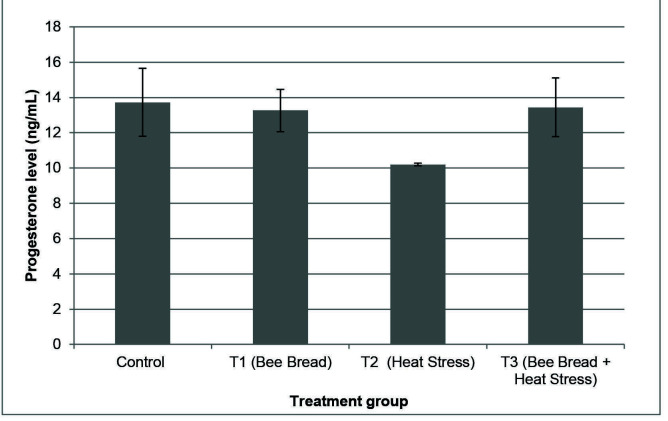
The progesterone level (mean ± SEM) in different treatment groups which are Control (C: standard feeding treatment), Treatment 1 (T1: Bee bread), Treatment 2 (T2: Heat stress) and Treatment 3 (T3: Bee bread + Heat stress).

**Figure 4:**
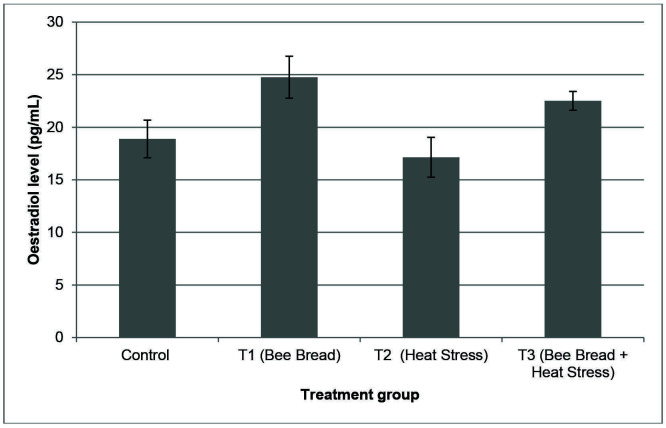
The oestradiol level (mean ± SEM) of in different treatment groups which are Control (C: standard feeding treatment), Treatment 1 (T1: Bee bread), Treatment 2 (T2: Heat stress) and Treatment 3 (T3: Bee bread + Heat stress).

Figs. 3 and 4 show the level of progesterone and estradiol levels in all experimental groups. However, no significant differences were observed in the progesterone and estradiol levels between all experimental groups.

The higher corticosterone hormone level in T2 group proved that the HPA axis was activated during exposure to heat stress. The HPA axis is one of the main endocrine components involved in the stress system response (Sheng *et al*. 2021). The activation of the HPA axis has resulted in the production of corticotropin-releasing hormone (CRH) and arginine-vasopressin (AVP). Both CRH and AVP stimulate the anterior pituitary to generate and release ACTH synergistically. Subsequently, the ACTH stimulates the adrenal cortex’s synthesis of glucocorticoids such as cortisol and corticosterone (Lucy 2019). In a rat’s adrenal cortex, corticosterone is one of the most abundant glucocorticoids produced (Ieko *et al*. 2019).

Rats in T2 group had a higher level of corticosterone than in the control and T3 groups which suggests that exposure to heat stress during pregnancy has resulted in increased corticosterone levels in dams of the present study. Similar findings were also reported by Wang *et al*. (2015), Zhang *et al*. (2019) and Sadek *et al*. (2021) showing that stressed dams had greater levels of plasma corticosterone than control dams. Previous research has demonstrated that high cortisol levels have a negative feedback effect on the production of corticotropin-releasing hormone (CRH) from the hypothalamus, causing the decrease of corticosterone release from the adrenal cortex (Allen & Sharma 2021). However, with prolonged stress, that negative feedback fails to limit cortisol release, and its levels rise to dangerously high levels, eventually resulting in neuroendocrine, cardiovascular and immunological diseases (Sadek *et al*. 2021).

According to Valsamakis *et al*. (2019), the HPA axis hormones have powerful, mostly inhibitory effects on female reproductive axis, the hypothalamic pituitary ovarian (HPO) axis. HPO axis is suppressed by restriction of GnRH production leading to inhibition of the release of LH by the pituitary (Wagenmaker & Moenter 2017). This was evident in the reduction of LH, FSH and estradiol secretion upon stressor exposure (El-Ratel *et al*. 2020). Therefore, these findings might suggest that higher corticosterone level due to stressor exposure including heat stress could potentially harm the reproductive activity in female which required further investigations.

Meanwhile, supplementation of bee bread during heat stress exposure (T3) has demonstrated a significant reduction in the corticosterone level suggesting that consumption of bee bread could minimise the stress effect on the body. The same findings were reported by Haron *et al*. (2014), showing that the level of corticosterone was reduced with the consumption of honey during stress in pregnant rats. According to Morsy *et al*. (2021), the presence of flavonoids in red propolis extract (RPE) may have contributed in lowering cortisol concentrations found in propolis-treated sheep. This can be rationalised by the function of antioxidants in honey or bee bread which are responsible in removing reactive oxygen and reactive nitrogen species, thereby protecting neurons in the brain from harm (Abdulmajeed *et al*. 2016). Bee bread is also rich in vitamins including vitamin C that ranged from 0.1087 to 0.1152 mg/g (Mohammad *et al*. 2020). A study found that supplementation of vitamin C in heat-stressed broiler has also resulted in lower corticosterone levels (del Barrio *et al*. 2020). This implies that bee bread supplementation was able to lower the corticosterone level possibly by the action of the antioxidant activity and its nutritional content that leads to better pregnancy outcomes

### Histological Analysis of Adrenal Gland

Table 1 shows the findings on the thickness of three zonas of the adrenal cortex. There is no significant difference was observed in the thickness of zona glomerulosa and zona reticularis among all experimental groups.

However, the thickness of zona fasciculata in T2 group was significantly higher when compared to control, T1, and T3 groups as shown in Table 1. This finding conforms with previous studies that found significant increase in the thickness of zona fasciculata layer of rat’s subjected to chronic unpredictable mild stress paradigm (CUMS) (Sadek *et al*. 2021). Zona fasciculata is the main source of glucocorticoids production such as corticosterone that being stimulated by adrenocorticotrophic hormone (ACTH) during stressful conditions (Levin *et al*. 2019; Sheng *et al*. 2021). Increase in the thickness of the zona fasciculata during stress may reflect its cell’s secretory activity in terms of generating and secreting corticosterone upon activation of the HPA axis. Another study by Zaki *et al*. (2018) also observed that stress induced changes in adrenal cortex by forming deformed irregulars capsule observed in both zona glomerulosa and zona fasciculata. Heat stress in T2 has altered normal functions of adrenal gland by increasing glucocorticoids to some extent thus resulting in a higher corticosterone level in T2 group of the present study. Although there is no significant difference was evident in the thickness of zona glomerulosa, however, the increased thickness of zona glomerulosa in T2 group might suggest its response in synthesising aldosterone for water and sodium maintenance during dehydration that may be caused by heat stress (Rakova *et al*. 2017) although no further analysis was undertaken in this study. This suggests that more research needs to be undertaken to determine the exact effect of heat stress on zona glomerulosa. The results also suggest that consumption of bee bread during heat stress by rats in T3 group has significantly reduced the thickness of zona fasciculata compared to the T2 group, reflecting the drop in corticosterone hormone in rats.

## CONCLUSION

In conclusion, exposure to heat stress significantly increased corticosterone levels. Heat stress also significantly increases the thickness of zona fasciculata which imply the cell’s secretory activity in terms of generating and secreting corticosterone upon activation of the HPA axis. Furthermore, supplementation of bee bread during heat stress significantly reduced the thickness of zona fasciculata and corticosterone levels. These findings may suggest that bee bread is effective as a neutralizer in lowering the production of stress hormone.

## Figures and Tables

**Table 1:** The thickness of zona glomerulosa, zona fasciculata and zona reticularis of rats’ adrenal gland.

Zona	Control	T1 (Bee bread)	T2 (Heat stress)	T3 (Bee bread + Heat stress)
Zona Glomerulosa (μm)	7.05 ± 0.21	6.85 ± 0.21	7.98 ± 0.40	7.51 ± 0.17
Zona Fasciculata (μm)	76.93 ± 3.36^a^	74.27 ± 4.31^a^	94.95 ± 1.55^b^	79.88 ± 3.08^a^
Zona Reticularis (μm)	45.83 ± 3.26	32.80 ± 2.46	30.42 ± 2.93	43.18 ± 6.34

*Notes*: Data are presented as mean ± SEM. Significant differences were determined by one-way ANOVA followed by Tukey’s post hoc test with *P* < 0.05. Different superscript (a, b) indicates a significant difference between the treatment groups.
